# CXCL17 Is a Specific Diagnostic Biomarker for Severe Pandemic Influenza A(H1N1) That Predicts Poor Clinical Outcome

**DOI:** 10.3389/fimmu.2021.633297

**Published:** 2021-02-26

**Authors:** Jose Alberto Choreño-Parra, Luis Armando Jiménez-Álvarez, Gustavo Ramírez-Martínez, Montserrat Sandoval-Vega, Citlaltepetl Salinas-Lara, Carlos Sánchez-Garibay, Cesar Luna-Rivero, Erika Mariana Hernández-Montiel, Luis Alejandro Fernández-López, María Fernanda Cabrera-Cornejo, Eduardo Misael Choreño-Parra, Alfredo Cruz-Lagunas, Andrea Domínguez, Eduardo Márquez-García, Carlos Cabello-Gutiérrez, Francina Valezka Bolaños-Morales, Lourdes Mena-Hernández, Diego Delgado-Zaldivar, Daniel Rebolledo-García, Parménides Guadarrama-Ortiz, Nora E. Regino-Zamarripa, Criselda Mendoza-Milla, Ethel A. García-Latorre, Tatiana Sofía Rodríguez-Reyna, Diana Cervántes-Rosete, Carmen M. Hernández-Cárdenas, Shabaana A. Khader, Albert Zlotnik, Joaquín Zúñiga

**Affiliations:** ^1^ Escuela Nacional de Ciencias Biológicas, Instituto Politécnico Nacional, Mexico City, Mexico; ^2^ Laboratorio de Inmunobiología y Genética, Instituto Nacional de Enfermedades Respiratorias “Ismael Cosío Villegas”, Mexico City, Mexico; ^3^ Facultad de Estudios Superiores Iztacala, Universidad Nacional Autónoma de México, Mexico City, Mexico; ^4^ Departamento de Neuropatología, Instituto Nacional de Neurología y Neurocirugía “Manuel Velasco Suarez”, Mexico City, Mexico; ^5^ Department of Pathology, Instituto Nacional de Enfermedades Respiratorias “Ismael Cosío Villegas”, Mexico City, Mexico; ^6^ Tecnologico de Monterrey, Escuela de Medicina y Ciencias de la Salud, Mexico City, Mexico; ^7^ Posgrado en Ciencias Biológicas, Universidad Nacional Autónoma de México, Mexico City, Mexico; ^8^ Department of Virology, Instituto Nacional de Enfermedades Respiratorias Ismael Cosío Villegas, Mexico City, Mexico; ^9^ Subdirection of Surgery, Instituto Nacional de Enfermedades Respiratorias Ismael Cosío Villegas, Mexico City, Mexico; ^10^ Departments of Dermatology and Education, Instituto Nacional de Ciencias Médicas y Nutrición Salvador Zubirán, Mexico City, Mexico; ^11^ Centro Especializado en Neurocirugía y Neurociencias México (CENNM), Mexico City, Mexico; ^12^Departamento de Fibrosis Pulmonar, Instituto Nacional de Enfermedades Respiratorias “Ismael Cosío Villegas”, Mexico City, Mexico; ^13^ Respiratory Critical Care Unit, Instituto Nacional de Enfermedades Respiratorias Ismael Cosío Villegas, Mexico City, Mexico; ^14^ Department of Molecular Microbiology, Washington University School of Medicine in St Louis, St. Louis, MO, United States; ^15^ Department of Physiology & Biophysics School of Medicine, Institute for Immunology, University of California, Irvine, CA, United States

**Keywords:** influenza A(H1N1), SARS-CoV-2, COVID-19, tuberculosis, chemokines, CXCL17

## Abstract

The C-X-C motif chemokine ligand 17 (CXCL17) is chemotactic for myeloid cells, exhibits bactericidal activity, and exerts anti-viral functions. This chemokine is constitutively expressed in the respiratory tract, suggesting a role in lung defenses. However, little is known about the participation of CXCL17 against relevant respiratory pathogens in humans. Here, we evaluated the serum levels and lung tissue expression pattern of CXCL17 in a cohort of patients with severe pandemic influenza A(H1N1) from Mexico City. Peripheral blood samples obtained on admission and seven days after hospitalization were processed for determinations of serum CXCL17 levels by enzyme-linked immunosorbent assay (ELISA). The expression of CXCL17 was assessed by immunohistochemistry (IHQ) in lung autopsy specimens from patients that succumbed to the disease. Serum CXCL17 levels were also analyzed in two additional comparative cohorts of coronavirus disease 2019 (COVID-19) and pulmonary tuberculosis (TB) patients. Additionally, the expression of CXCL17 was tested in lung autopsy specimens from COVID-19 patients. A total of 122 patients were enrolled in the study, from which 68 had pandemic influenza A(H1N1), 24 had COVID-19, and 30 with PTB. CXCL17 was detected in *post-mortem* lung specimens from patients that died of pandemic influenza A(H1N1) and COVID-19. Interestingly, serum levels of CXCL17 were increased only in patients with pandemic influenza A(H1N1), but not COVID-19 and PTB. CXCL17 not only differentiated pandemic influenza A(H1N1) from other respiratory infections but showed prognostic value for influenza-associated mortality and renal failure in machine-learning algorithms and regression analyses. Using cell culture assays, we also identified that human alveolar A549 cells and peripheral blood monocyte-derived macrophages increase their CXCL17 production capacity after influenza A(H1N1) pdm09 virus infection. Our results for the first time demonstrate an induction of CXCL17 specifically during pandemic influenza A(H1N1), but not COVID-19 and PTB in humans. These findings could be of great utility to differentiate influenza and COVID-19 and to predict poor prognosis specially at settings of high incidence of pandemic A(H1N1). Future studies on the role of CXCL17 not only in severe pandemic influenza, but also in seasonal influenza, COVID-19, and PTB are required to validate our results.

## Introduction

The C-X-C motif chemokine ligand 17 (CXCL17) is a mucosal chemokine expressed in the respiratory tract under both homeostatic and inflammatory conditions (1–3). Its homeostatic functions include the recruitment of various myeloid cell populations [dendritic cells (DCs), monocytes, and macrophages] to mucosal tissues, including the lung (4). Under inflammatory conditions, its functions are less well characterized, although it has been reported to promote anti-inflammatory activities and likely prevent autoimmunity (2, 4). CXCL17 also exhibits broad antimicrobial activity (2, 4), suggesting that it may play an important role in protective immunity against respiratory pathogens. In viral infections, there is only a report suggesting that CXCL17 participates in immunity against genitourinary herpes infections (5). However, despite CXCL17 being one of the most highly expressed chemokines in the human lung, its potential role in human respiratory infections has not been studied.

Here, we report high levels of CXCL17 in serum from patients with acute respiratory distress syndrome (ARDS) associated with pandemic influenza A(H1N1). Conversely, serum CXCL17 levels in severely ill coronavirus disease 2019 (COVID-19) patients and individuals with pulmonary tuberculosis (PTB) were not elevated, indicating that CXCL17 serum levels are a specific diagnostic marker that differentiate pandemic influenza A(H1N1) from other infections in patients with respiratory illness. We also analyzed the pattern of expression of this chemokine in lung specimens from patients with influenza and COVID-19. Importantly, analyses of CXCL17 serum levels in influenza A (H1N1) patients revealed that high levels of CXCL17 correlated with poor prognosis, including kidney injury and death. Finally, we identified that human alveolar A549 cells and peripheral blood monocyte-derived macrophages produce CXCL17 after an *in vitro* exposure to the influenza A(H1N1) pdm09 virus.

Our data might be helpful for the clinical decision-making process during the ongoing flu season, which is projected to be one of the most challenging public health crises in recent history due to the convergence of influenza and COVID-19. Moreover, our results for the first time indicate that CXCL17 might have a prognostic value during severe pandemic influenza A(H1N1). Overall, these preliminary data justify future efforts to address a possible role for CXCL17 during influenza. Also, future studies on the role of CXCL17 in COVID-19 and PTB are warranted.

## Materials and Methods

### Study Design and Participants

We conducted a cohort study in hospitalized patients (N = 68) with laboratory-confirmed influenza A(H1N1)pdm09 virus infection (hereinafter referred to as influenza) that attended the emergency department of the National Institute for Respiratory Diseases (INER) of Mexico during the 2018/19 and 2019/20 flu seasons. Patients with influenza-like illness (ILI) that progressed to ARDS requiring mechanical ventilation (MV) and admission to the intensive care unit (ICU) were eligible. These subjects were screened for influenza using a rapid influenza diagnostic test (RIDT; Fuji dri-chem immuno AG cartridge FluAB kit, Fujifilm Corp, Tokyo, Japan) in fresh respiratory swab specimens. Simultaneously, further molecular characterization of the causative influenza virus subtype was assessed by RT-PCR, as previously described (6).

In addition, we included a group of patients with COVID-19 (N = 24) that attended INER or the National Institute of Medical Sciences and Nutrition (INCMNSZ) in Mexico City, from March to May of 2020. The infection with SARS-CoV-2 was detected by RT-PCR, as described below (7). We also enrolled a third cohort of individuals with active PTB (N = 30) recruited at the “Tuberculosis Clinic” of the INER in Mexico City. The diagnosis of PTB was performed by direct clinical examination, radiological evaluation, and microbiological analyses of sputum specimens, as described before (8). None of the patients enrolled in the study had human immunodeficiency virus (HIV) infection. We retrieved clinical and demographic data from the participants. Peripheral blood samples were taken from each patient within the first 24 h following hospital admission to determine serum CXCL17 levels. An additional blood sample was obtained from patients with influenza seven days after admission. Healthy volunteer individuals (N = 30) were also recruited to participate in the study and served as controls.

### RT-PCR for SARS-CoV-2 Detection

Briefly, viral RNA was extracted from clinical samples with the MagNA Pure 96 system (Roche, Penzberg, Germany). The RT-PCR reactions were performed in a total volume of 25 μl, containing 5 μl of RNA, 12.5 μl of 2× reaction buffer provided with the Superscript III one-step RT-PCR system with Platinum Taq Polymerase (Invitrogen, Darmstadt, Germany; containing 0.4 mM of each deoxyribose triphosphates (dNTP) and 3.2 mM magnesium sulfate), 1μl of reverse transcriptase/Taq mixture from the kit, 0.4 μl of a 50 mM magnesium sulfate solution (Invitrogen), and 1 μg of non-acetylated bovine serum albumin (Roche). Primer and probe sequences, as well as optimized concentrations, are shown in **Supplementary Table 1**. All oligonucleotides were synthesized and provided by Tib-Molbiol (Berlin, Germany). Thermal cycling was performed at 55°C for 10 min for reverse transcription, followed by 95°C for 3 min and then 45 cycles of 95°C for 15 s, 58°C for 30 s.

### *In Vitro* Infection Assays With Influenza A(H1N1) pdm09 Virus

Influenza A(H1N1) pdm09 virus was isolated from patients with severe pneumonia in Madin–Darby canine kidney cells (MDCK). Virus infectivity was assessed by titration of tissue culture infection dose 50% (TCID_50_) in MDCK cells, as previously described (9). Human lung adenocarcinoma epithelial cells A549 were purchased from the American Type Culture Collection (ATCC, Rockville, MD) and cultured in DMEM with 10% fetal bovine serum (Lonza) at 37°C and 5% CO_2_. Human macrophages were obtained and cultured as described before (9). A549 epithelial cells and macrophages were infected with influenza A(H1N1) pdm09 virus at a multiplicity of infection (MOI) of 5 for one hour. Mock-treated cells received a virus-free culture medium. After incubation, cells were washed twice with phosphate buffer saline (PBS). Media containing the influenza virus was replaced with a virus-free culture medium. Supernatants were collected after 24, 48, and 72 h, for CXCL17 quantitation.

### CXCL17 Levels

Levels of CXCL17 in human serum and culture supernatants were determined by enzyme-linked immunosorbent assay (ELISA) using a commercial kit (MBS916471, My BioSource, USA) following the manufacturer’s instructions.

### Histological Analysis and Immunohistochemistry

Formalin-fixed and paraffin-embedded lung autopsy specimens from patients who died of influenza or COVID-19 (N = 2 patients per group) were obtained from the Pathology Department of the INER. Sections of 3–5 μm were processed for hematoxylin–eosin (H&E) staining for histopathological analysis. For immunohistochemistry (IHQ), lung sections were mounted on silane-covered slides, deparaffinized, and the endogenous peroxidase blocked. Sections were incubated overnight at room temperature with an optimal dilution (1:100) of the Mouse Anti-Human CXCL17/VCC-1 Monoclonal Antibody (Clone # 422208, MAB4207, R&D Systems, Minneapolis, MN). Secondary biotinylated antibodies labeled with peroxidase were added, and those attached were revealed with diaminobenzidine (ImmunoCruz™ rabbit ABC Staining System, Santa Cruz Biotechnology, Santa Cruz, CA). Slides were counter-stained with hematoxylin.

### Ethics Statement

The current study was reviewed and approved by the Institutional Review Boards of the INER (approval number: B04-15, B28-16 and B09-20) and the INCMNSZ (approval number: 3349) in Mexico City. All participants or their legal guardians provided written consent to participate in the study. Serum samples were managed according to the Mexican law NOM-012-SSA3-2012, which establishes the criteria for the execution of clinical investigations in humans.

### Statistical Analysis

Descriptive statistics were used to characterize the population under study clinically. Frequencies and proportions were calculated for categorical data. Means, medians, standard deviations (SD), interquartile ranges (IQR), and 95% confidence intervals (CI) were used for continuous variables. Differences in categorical variables between groups were assessed by the Fisher exact or Chi-square test, as appropriate. For comparisons of continuous variables between two groups, we used the Mann–Whitney U test. For differences of continuous data between more than two groups, we used the Kruskal–Wallis test with *post hoc* Dunn test. Differences in serum levels of CXCL17 measured in serial samples were determined using the Wilcoxon matched-pairs signed-rank test. Multiple linear regression analyses using Spearman rank correlation coefficients were used to determine correlations between continuous clinical variables and serum levels of CXCL17. The K-means algorithm was used for clustering study participant characteristics according to their diagnosis (influenza or COVID-19) and clinical outcome (survival or fatality). Before data visualization, clinical features and laboratory parameters were scaled and centered.

To identify the variables with the highest impact on disease diagnosis (influenza, COVID-19) and adverse outcomes (death), random forest analyses of 500 classification and regression trees (CARTs) were performed. The diagnostic accuracy of serum CXCL17 levels and other selected variables identified by random forest logarithm was further evaluated by bivariate logistic regression and Receiver Operating Characteristic (ROC) curve (AUC) analyses. In addition, Kaplan–Meier curves were constructed to look for differences in survival according to serum CXCL17 levels dichotomized by the ROC curve threshold with the highest diagnostic accuracy estimated using the Youden index. For random forest and logistic regression analyses, patients with any missing value in the variables of interest were excluded.

All analyses were conducted using GraphPad Prism 8 (La Jolla, CA) and R Statistical Software (Foundation for Statistical Computing, Vienna, Austria). Specific analytical tests are also described in the figure legends. Values of *p ≤*0.05 were considered as significant: *p ≤ 0.05, **p ≤ 0.01, ***p ≤ 0.001, ****p ≤ 0.0001.

## Results

### Participant Characteristics

A total of 68 patients infected with influenza were enrolled in the study. Their clinical characteristics are summarized in **Table 1**. Seventy percent of influenza patients were male, with a median age of 48 years. We also recruited 24 patients with COVID-19, from which most were males (75%), with a median age of 50 years. Most patients with influenza presented similar characteristics than COVID-19, except for some specific differences. For instance, obesity was more common among influenza patients, while the prevalence of other comorbidities did not differ between both diseases. Cough, dyspnea, fever, myalgia, and arthralgia were the most frequent symptoms of respiratory illness in both participant groups. Interestingly, dyspnea, rhinorrhea, and sputum production were more common during influenza, whereas dry cough and vomit were more frequent among COVID-19 patients (**Table 1**).

**Table 1:** Clinical characteristics and laboratory parameters of study participants.

Characteristics	Influenza, N = 68A	COVID-19, N = 24B	*p*-value, A *vs* B	PTB, N = 30C	*p*-value, A *vs* C
**Age (years), median (range)**	48 (20–77)	50 (28–73)	0.6536	39 (20–46)	0.0010
**Males**	48 (70.5)	18 (75)	0.7953	20 (66.6)	0.8126
**BMI**	33.4 (30–38.2)	28.9 (25.2–30.3)	<0.0001	20.6 (18.8–23.1)	<0.0001
**Comorbidities**					
Smoking	27 (39.7)	5 (20.8)	0.1349	0 (0)	<0.0001
Diabetes	14 (20.5)	7 (29.1)	0.4063	11 (36.6)	0.1306
SAH	18 (26.4)	5 (20.8)	0.7848	2 (6.6)	0.0295
OSA	4 (5.8)	0 (0)	0.5695	0 (0)	0.3095
COPD	3 (4.4)	1 (4.1)	>0.9999	0 (0)	0.5507
**Symptoms at onset**					
Fever	62 (91.1)	18 (75)	0.0725	–	–
Myalgia	56 (82.3)	18 (75)	0.5500	–	–
Arthralgia	53 (79.1)	16 (66.6)	0.2847	–	–
Headache	33 (48.52)	11 (45.8)	>0.9999	–	–
Dyspnea	65 (95.5)	16 (66.6)	0.0007	–	–
Nasal congestion	13 (19.1)	1 (4.1)	0.1038	–	–
Rhinorrhea	26 (38.2)	3 (12.5)	0.0221	–	–
Sore throat	24 (35.8)	4 (16.6)	0.1223	–	–
Thoracic pain	9 (13.2)	0 (0)	0.1053	–	–
Cough	66 (97)	21 (87.5)	0.1093	–	–
Sputum	38 (55.8)	2 (8.3)	<0.0001	–	–
Dry cough	27 (40.2)	19 (79.1)	0.0017	–	–
Fatigue	49 (72)	18 (75)	>0.9999	–	–
Diarrhea	5 (7.35)	5 (20.8)	0.1196	–	–
Nausea	4 (5.88)	3 (12.5)	0.3717	–	–
Vomit	2 (2.9)	4 (16.6)	0.0382	–	–
**Illness onset - admission (days)**	7.5 (5.2–12)	6 (4.2–10.7)	0.3756	–	–
**Admission vital signs**					
Body temperature (°C)	38 (37–38)	37 (37–37.08)	0.0018	–	–
Respiratory rate (bpm)	25 (20–30)	24 (22–26)	0.3486	–	–
Hearth rate (bpm)	96 (85-108)	85 (75–96)	0.0074	–	–
MAP (mmHg)	85.4 (75–118.3)	77.5 (71–87)	0.0677	–	–
**Glucose (mg/dl)**	144.9 (114.9–221.4)	115.6 (96.7–200.7)	0.0771	–	–
**Blood count**					
White blood cells (10^9^/L)	7.2 (5.6–10.3)	9.1 (5.2–12.2)	0.4429	–	–
Neutrophils (10^9^/L)	5.7 (4.5–8.3)	7.4 (3.6–10.1)	0.7057	–	–
Lymphocytes (10^9^/L)	0.8 (0.5–1.1)	0.8 (0.6–1.0)	0.4657	–	–
NLR	8.5 (5.4–12.6)	8.7 (3.9–13.4)	0.7927	–	–
Hgb (g/dl)	14.9 (13.3–17.3)	14.1 (13.2–15.3)	0.1015	–	–
Platelets (10^9^/L)	177.5 (136.5–216.5)	202 (145.8–256.8)	0.2523	–	–
**Renal function**					
Cr (mg/dL)	1.02 (0.7–1.4)	1.0 (0.8-1.4)	0.8754	–	–
BUN (mg/dl)	24.9 (15.5–35.7	18.3 (13.6-26.8)	0.1390	–	–
Na (mmol/L)	137.4 (133.4–140.5)	138.6 (136-141.5)	0.2155	–	–
K (mmol/L)	4.1 (3.8–4.5)	4.1 (3.9-4.3)	0.9384	–	–
**Liver function**					
Total bilirubin (mg/dl)	0.6 (0.4–0.8)	0.4 (0.3-0.7)	0.0900	–	–
AST (U/L)	61.8 (44.7–87)	35.1 (23.6-80.7)	0.0055	–	–
ALT (U/L)	41 (26.3–52.2)	32.7 (25.1-48.4)	0.2768	–	–
**Other biomarkers**					
LDH (U/L)	643.8 (500–877.9)	341.7 (251.5-477.7)	<0.0001	–	–
ALP (U/L)	121.7 (98.2–161.1)	78 (67.7-88.2)	<0.0001	–	–
CPK (U/L)	274.4 (108–700.8)	117.8 (64-117.8)	0.4754	–	–
Procalcitonin (ng/ml)	0.55 (0.15–1.92)	0.1 (0.05-0.17)	<0.0001	–	–
**PaO_2_/FiO_2_**	82.5 (59.9–143.5)	127.8 (94.7-198.1)	0.0031	–	–
**Severity of illness**					
SOFA	7 (5–9)	5 (3-6)	0.0002	–	–
APACHE II	10 (7–16)	7 (4-8)	0.0069	–	–
**Respiratory support**					
High flow nasal cannula	0 (0)	7 (29.1)	<0.0001	0 (0)	>0.9999
MV	68 (100)	17 (70.8)	<0.0001	0 (0)	<0.0001
Prone position	40 (58.8)	8 (33.3)	0.0359	0 (0)	<0.0001
ECMO	7 (10.2)	0 (0)	0.1836	0 (0)	0.1836
**Renal replacement therapy**	16 (23.5)	2 (8.3)	0.1399	0 (0)	0.0022
**Mortality**	16 (23.5)	10 (41.6)	0.1151	0 (0)	0.0022

Data are displayed as n (%) or median (IQR). N is the total number of patients with available data. ALP, alkaline phosphatase; APACHE-II, Acute Physiology And Chronic Health Evaluation II; AST, aspartate aminotransferase; ALT, alanine aminotransferase; BMI, body mass index; bpm, breaths/beats per minute; BUN, blood ureic nitrogen; COPD, chronic obstructive pulmonary disease; CPK, creatine phosphokinase; Cr, creatinine; ECMO, extra-corporeal membrane oxygenation; FiO2, fraction of inspired oxygen; HCO3, bicarbonate; Hgb, hemoglobin; IQR, interquartile range; ICU, intensive care unit; LDH, lactate dehydrogenase; MAP, mean arterial pressure; MV, mechanical ventilation; ND, not determined; NLR, neutrophil/lymphocyte ration; OSA, obstructive sleep apnea syndrome; PaO2, partial pressure of oxygen in arterial blood; PCO2, partial pressure of carbon dioxide in blood; SAH, systemic arterial hypertension; SD, standard deviation; SOFA, Sequential Organ Failure Assessment. Differences in continuous variables were estimated using the Mann–Whitney U test. Differences in categorical variables were calculated using the Fisher’s exact or the Chi square test as appropriate.

Triage vital signs were similar between groups, except for a higher blood temperature and heart rate in influenza patients. Also, most laboratory parameters routinely tested in emergency departments did not differ between individuals with influenza and COVID-19. The levels of aspartate aminotransferase (AST), lactate dehydrogenase (LDH), alkaline phosphatase (ALP), and procalcitonin were increased in influenza patients as compared to COVID-19 subjects (**Table 1**). Similarly, severity of illness scores at admission, including the Sequential Organ Failure Assessment (SOFA), and the Acute Physiology and Chronic Health Evaluation II (APACHE-II), were higher in influenza cases. We should note that despite this, COVID-19 patients showed higher mortality compared to influenza (41 *vs*. 23%, *p* = 0.1151).

An additional cohort of 30 patients with active PTB were included in the study. These individuals were sex-matched with influenza patients. However, they presented some differences regarding their clinical and demographic characteristics. First, PTB patients were younger than influenza subjects, with a median age of 38 years. Sixty-six percent were males and showed a lower body mass index (BMI) than influenza and COVID-19 patients. There were no differences in the prevalence of diabetes between groups. Finally, although PTB showed a milder and chronic clinical disease with regards to influenza and COVID-19, they had high degrees of lung damage according to their scores in a quantitative scale that evaluates changes on chest radiographs (data not shown) (10).

### Expression of CXCL17 in the Lung of Patients With Influenza or COVID-19

**Figure 1:**
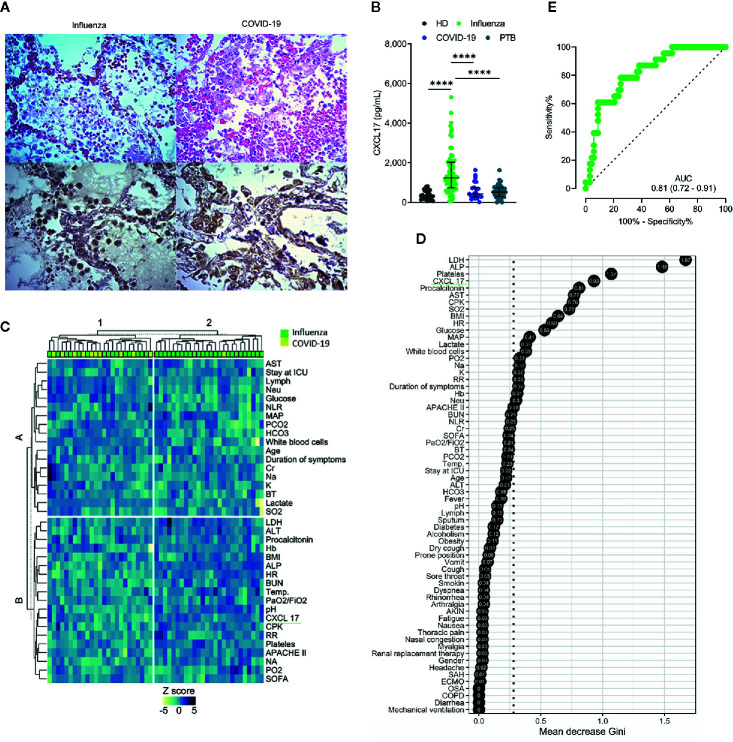
CXCL17 in the lung and serum of influenza, COVID-19, and PTB patients. **(A)** Morphological changes observed in the lung of influenza and COVID-19 patients (n = 2 per group). H&E, ×400. The expression of CXCL17 was analyzed in lung autopsy specimens from influenza (left lower panel) and COVID-19 (right lower panel). IHQ, ×400. **(B)** Serum CXCL17 levels in healthy donors (HD; n = 30) patients with influenza (n = 68), COVID-19 subjects (n = 24), and PTB patients (n = 30). Kruskal–Wallis test and *post hoc* Dunn test (plots display medians with interquartile ranges; ****p ≤ 0.0001). **(C)** K-means clustering analysis of the clinical characteristics of influenza and COVID-19 patients. **(D)** Random forest algorithm showing the most important factors that differentiate influenza from COVID-19. The points represent the mean decrease Gini values, indicative of the importance of each variable with respect to the mean importance of the model (discontinuous vertical line). **(E)** Receiver operating characteristic (ROC) curve of the levels of CXCL17 in influenza and COVID-19 subjects. The graph displays area under the curve (AUC) and 95% CI interval values.

CXCL17 is constitutively expressed in the respiratory tract and lungs of mice and humans (1, 2). Under inflammatory conditions, the production of this chemokine is known to be further upregulated (2, 4, 5, 11, 12). To investigate whether CXCL17 may participate in immunity against the influenza A(H1N1) virus, we analyzed the tissue expression pattern of CXCL17 in lung autopsy specimens obtained from patients who died of influenza. The histological changes induced in the lungs during influenza were mainly characterized by intra-alveolar inflammatory infiltrates consisting of macrophages and polymorphonuclear cells scattered between areas of edema, hemorrhage, and fibrin deposits (**Figure 1A**, left upper panel). We also noted that the integrity of alveolar walls and the micro-architecture of the lung were conserved in influenza patients (**Supplementary Figure 1**). CXCL17 expression was detected mainly within the cytoplasm of infiltrating macrophages, but not in polymorphonuclear cells. The expression of CXCL17 was also detected within alveolar epithelial cells (**Figure 1A**, left lower panel). Blood vessels and pleura did not express CXCL17 in lung autopsy specimens from influenza patients (data not shown).

We also evaluated the tissue-expression pattern of CXCL17 in lungs of patients that succumbed to COVID-19. Interestingly, we found that SARS-CoV-2 induced distinctive morphological changes in the infected lung, characterized by an intense inflammatory infiltrate affecting extensive areas of the parenchyma, as well as the thickness of alveolar walls, and partial loss of the histological architecture of the lung (**Supplementary Figure 1** and **Figure 1A**, right upper panel). These changes are compatible with interstitial pneumonia. As observed in influenza infected lung sections, the expression of CXCL17 in the lungs of deceased COVID-19 patients was also detected within macrophages and alveolar epithelial cells (**Figure 1A**, right lower panel). CXCL17 was not expressed in endothelial cells of alveolar capillaries, but pleura showed increased CXCL17 expression (data not shown). Collectively, these findings demonstrate that CXCL17 can be detected in the lungs during influenza and SARS-CoV-2 infection.

### High Serum Levels of CXCL17 Distinguish Influenza From Other Respiratory Infections

Although CXCL17 is mainly produced at mucosal surfaces, increased serum levels of this chemokine might serve as a readout of active local immune responses. Thus, we addressed whether the CXCL17 expression found in the lungs of influenza- and SARS-CoV-2-infected patients could be also detected in the serum. Our results indicate that CXCL17 levels were significatively elevated in the serum of influenza cases, but not in healthy donors (HD) or COVID-19 subjects. The latter group indeed showed low serum CXCL17 levels, similar to the levels observed in HD (**Figure 1B**). These findings contrast with the expression of CXCL17 detected in the lung of COVID-19 patients, suggesting that the levels of CXCL17 in serum and its expression in lung tissue specimens do not correlate during the course of the disease. Nonetheless, our lung immunohistochemical analyses only focused on the expression of this chemokine in the late stages of influenza and COVID-19, whereas serum samples were taken within the first day after patients´ hospital admission. We also measured the levels of CXCL17 in serum samples from PTB patients. Notably, we found that the levels of the chemokine in PTB individuals were much lower compared to those observed in influenza patients (**Figure 1B**). In contrast, no differences in serum CXCL17 levels were observed between COVID-19 and PTB patients. These findings suggest distinctive serum CXCL17 dynamics during influenza, COVID-19, and PTB that could potentially be harnessed for diagnostic purposes. This is important since influenza and COVID-19 will converge at some point during the ongoing winter in the North Hemisphere. As such, physicians will require novel rapidly testable diagnostic biomarkers to discriminate both diseases, specially at setting of limited availability of RT-PCR tests.

Hence, next we investigated if CXCL17, along with other clinical characteristics, could have certain diagnostic value to discriminate between both viral infections. In an unsupervised clustering analysis, we found that some influenza patients grouped together according to their clinical and laboratory parameters, but another cluster was formed by combined influenza and COVID-19 subjects (**Figure 1C**). This suggests that the differentiation of the two infections by clinical characteristics would be difficult in the emergency room. Nonetheless, in a random forest analysis, CXCL17 was among the most explicative variables of the viral subtype (**Figure 1D**). Indeed, in a bivariate logistic regression analysis using the variables identified in the random forest algorithm, only CXCL17, along with procalcitonin, showed significant association with influenza (**Supplementary Figure 2**). LDH and ALP were marginally associated with influenza, whereas platelets showed no correlation with any type of infection. Interestingly, although irrelevant in the random forest algorithm, symptoms such as dyspnea, rhinorrhea, and sputum production were predictors of influenza, whereas dry cough and vomit were associated with COVID-19.

To further estimate the diagnostic value of CXCL17, we performed a ROC curve analysis with the serum levels of CXCL17 of influenza and COVID-19 subjects. We observed that CXCL17 levels could reliably differentiate between influenza and COVID-19, with an AUC of 0.81 (**Figure 1E**). Using a cut-off value of 841 pg/ml, elevated serum levels of this chemokine have a 78.2% sensitivity, 73.5% specificity, 89.2% PPV, 51.3% NPV, and an OR of 8.79 (3.1–26, 95% CI) to distinguish influenza from COVID-19. Together, our results point to the diagnostic use of serum CXCL17 levels in patients with acute respiratory illness to enable distinguishing infection between these viruses, although these findings must be validated in larger cohorts of patients, as well as in patients infected with seasonal influenza viruses.

### Dynamics and Prognostic Value of Serum CXCL17 Levels in Influenza

**Figure 2:**
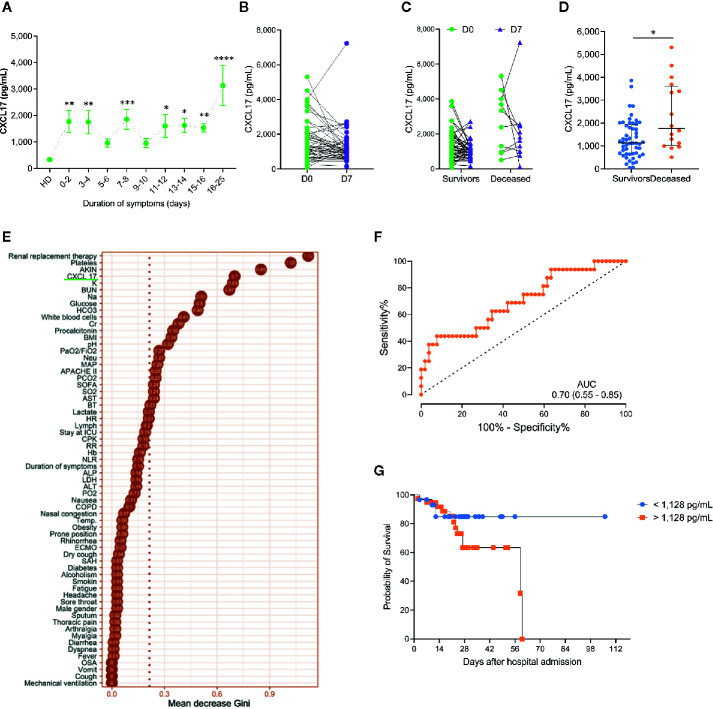
Dynamics and prognostic value of serum CXCL17 levels in influenza **(A)** Influenza patients (n = 68) were grouped according to their duration of symptoms on admission. Levels of CXCL17 were compared to healthy donors (HD; Kruskal–Wallis test and *post hoc* Dunn test; graph displays means and the standard error ( ± SE); *p ≤ 0.05, **p ≤ 0.01, ***p ≤ 0.001, ****p ≤ 0.0001). **(B)** We obtained a second serum sample in 54 influenza subjects seven days (D7) after hospital admission (D0). Levels of CXCL17 at D0 and D7 were compared with the Mann–Whitney U test. **(C)** Longitudinal change in CXCL17 levels in survivor and deceased influenza patients (Wilcoxon test). **(D)** Initial serum CXCL17 levels in survivor (n = 52) and deceased (n = 16) influenza patients (Mann–Whitney U test; plots display medians and interquartile ranges). **(E)** Random forest algorithm showing the most important factors that impact on influenza-associated mortality. **(F)** Receiver operating characteristic (ROC) curve of the levels of CXCL17 in survivor and deceased influenza subjects. The graph displays area under the curve (AUC) and 95% CI interval values. **(G)** Survival curves of influenza patients grouped according to their serum CXCL17 levels were compared with the log-rank test.

Next, we evaluated the dynamics of serum CXCL17 levels during influenza. For this purpose, we grouped influenza patients according to the duration of their illness, defined as the period from symptom onset to hospital admission. Interestingly, we found that levels of CXCL17 increased early in influenza patients within the first two days following the onset of symptoms, and levels remained increased during the first two weeks of illness. However, the maximum levels of CXCL17 were observed among influenza patients seeking medical attention three weeks after the onset of symptoms (**Figure 2A**). In 54 of the 68 influenza patients enrolled in the study, an additional serum sample was obtained seven days (D7) following hospital admission (D0). Although there were no differences in CXCL17 between D0 and D7, most patients showed constant or decreasing chemokine levels (**Figure 2B**), except for one individual who showed a notable increase in CXCL17 and succumbed to the infection. Overall, the dynamics of CXCL17 levels post-hospital admission were similar in survivors and deceased influenza patients (**Figure 2C**).

Importantly, serum levels of CXCL17 at D0 were significatively higher in patients who died of influenza as compared to survivors (**Figure 2D**). Indeed, CXCL17 again was among the variables with the higher importance for influenza-associated mortality in a random forest analysis (**Figure 2E**), showing an AUC of 0.70 to differentiate both groups in the ROC curve analysis (**Figure 2F**). Serum levels of CXCL17 above 1,128 pg/ml predicted a fatal outcome with a 75% sensitivity and 50% specificity, showing a non-significant OR value for mortality of 3.0 (0.86–9.24, 95% CI). The survival of patients with CXCL17 below such cut-off value was lower at day 14 after admission when compared to individuals with higher levels of this chemokine (**Figure 2G**, **Table 2**). Conversely, accumulated survival at days 28 and 60 following hospital admission was lower in influenza patients with serum levels of CXCL17 ≥1,128 pg/ml.

**Table 2:** Cumulative survival rates in patients with influenza according with their serum CXCL17 levels.

Time after hospital admission	Survival (%, 95% CI)	
	CXCL17 <1,128 pg/ml	CXCL17 ≥ 1,128 pg/ml
7 days	96.66 (78.6–99.52)	94.73 (80.55–98.65)
14 days	84.86 (64.38–94.06)	91.776 (76.56–97.27)
21 days	84.86 (64.38–94.06)	85.06 (67.5–94.16)
28 days	84.86 (64.38–94.06)	63.33 (41.48–78.89)
60 days	84.86 (64.38–94.06)	31.66 (1.93-71.53)

Survival rates and their 95% CI were estimated using the Kaplan-Meyer method and the log rank test.

**Figure 3:**
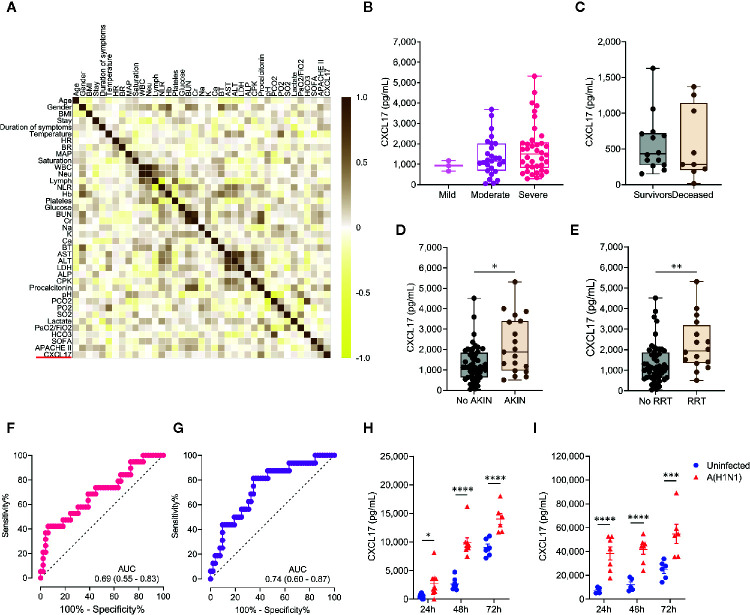
Extended predictive value of serum CXCL17 levels in influenza patients and cellular sources. **(A)** Multiple correlation analysis of the clinical variables and serum CXCL17 levels of influenza patients (n = 68). The heat color map gradient was constructed using Spearman R values. **(B)** Serum CXCL17 levels in influenza patients according to the severity of their acute respiratory distress syndrome (ARDS) on admission (mild, PaO2/FiO2 >200, n = 3; moderate, PaO2/FiO2 100–200, n = 27; severe, PaO2/FiO2 <100, n = 38; Kruskal–Wallis test and *post hoc* Dunn test). **(C)** Comparison of the levels of CXCL17 between COVID-19 patients that survived (n = 14) or died (n = 10). Mann–Whitney U test. **(D)** Serum levels of CXCL17 were compared between influenza patients that developed acute kindly injury (AKIN, n = 19), and individuals with normal renal function (n = 49). **(E)** Differences in CXCL17 levels between influenza patients according to their need for renal replacement therapy (RRT; n = 52 *vs* 16). **(B**–**E)** Plots display medians and interquartile ranges. Mann–Whitney U test. **(F)** Receiver operating characteristic (ROC) curve of the CXCL17 levels in influenza patients that developed AKIN and those that maintained normal renal function. The graph display area under the curve (AUC) and 95% CI interval values. **(G)** ROC curve analysis of the serum CXCL17 levels in patients requiring RRT. **(H)** Human A549 alveolar epithelial cells and **(I)** peripheral blood-monocyte derived macrophages were infected with a clinical isolate of the influenza A(H1N1) pdm09 virus for 24, 48, and 72 h n = 6–7 per group per time point). Levels of CXCL17 in supernatants from infected and uninfected cells were compared using the unpaired Student’s t test (n = 5 to 9 per group) at each time point. Values of *p* were corrected for multiple comparisons using the Holm–Sidak method. The data represent mean ( ± SE) values from 2 to 3 independent experiments per time point and experimental condition. *p ≤ 0.05, **p ≤ 0.01, ***p ≤ 0.001, ****p ≤ 0.0001.

Most clinical and laboratory parameters did not differ between the two groups of influenza patients (**Supplementary Table 2**). Furthermore, none of these clinical characteristics impacted the serum levels of CXCL17 (**Figure 3A**), and this chemokine was not associated with the severity of ARDS in terms of the PaO2/FiO2 values at admission (**Figure 3B**). These results together suggest that CXCL17 represents an independent prognostic factor for mortality in influenza. In contrast, no differences in serum CXCL17 levels at admission were observed between survivor- and deceased-COVID-19 patients (**Figure 3C**), and this chemokine showed no correlation with the lung damage score of patients with PTB (**Supplementary Figure 3**).

We also noted that CXCL17 was increased among influenza patients that developed acute kidney injury (AKIN; **Figure 3D**), and its levels were even higher in individuals requiring renal replacement therapy (**Figure 3E**). Using the same cut-off value of 1,128pg/mL, elevated serum levels of CXCL17 showed a 73.6% sensitivity, 51% specificity, and a non-significant OR of 2.91 (0.96–8.28, 95% CI) to predict the development of AKIN (**Figure 3F**). Similarly, increased levels of CXCL17 showed an 87.5% sensitivity, 53.8% specificity, and a significant OR of 8.16 (1.71–38, 95% CI) to predict the need for renal replacement therapy (**Figure 3G**).

### Influenza A(H1N1) pdm09 Virus Induces the Production of CXCL17 in Human Lung Epithelial Cells and Macrophages

The higher levels of CXCL17 in sera from influenza patients prompted us to investigate the possible cellular sources of CXCL17 during influenza. To this end, we infected human A549 lung alveolar epithelial cells and peripheral blood monocyte-derived macrophages with a clinical isolate of the influenza A(H1N1) pdm09 virus. Interestingly, while both human cell types produced high amounts of CXCL17 at 24 h, 48h, and 72h after the infection, A549 epithelial cells produced lower levels of CXCL17 in response to influenza as compared to macrophages (**Figures 3H, I**). These findings are consistent with the expression of CXCL17 in lung macrophages and alveolar epithelial cells in autopsy specimens from our influenza patients. This suggests that the influenza A(H1N1) pdm09 virus stimulates CXCL17 expression in humans, both *in vivo* and *in vitro*. However, further analyses are required to confirm a role for CXCL17 in influenza.

## Discussion

The clinical spectrum of ARDS encompasses very severe forms of the disease characterized by a profound respiratory failure. These manifestations are often related to dysregulated immune reactions elicited by different insults. The quality and magnitude of such responses may vary according to the causative agent of the illness. Thus, particular immune profiles of local and circulating cytokines may serve as readouts to differentiate between specific causes of ARDS. Here, we demonstrate that CXCL17 can be detected in post-mortem human lung specimens from patients with pandemic influenza A(H1N1) and COVID-19. Furthermore, serum levels of CXCL17 are differentially regulated during influenza and COVID-19, pointing to a possible diagnostic value for the chemokine. Importantly, we found that CXCL17 is not elevated in the serum of PTB patients neither. Although individuals with this disease rarely present with ARDS, the fact that only influenza but not PTB or COVID-19 patients showed high CXCL17 levels remarks the specific diagnostic potential of this chemokine during influenza.

Chemokines can be divided into homeostatic or inflammatory depending on their expression patterns (13). CXCL17 is considered a “dual” chemokine because it exhibits characteristics of both homeostatic and inflammatory chemokines. It is constitutively expressed in the mucosa of the respiratory tract with potential functions in lung defenses (14) and is known to chemoattract macrophages (4). This chemokine was the last member of the C-X-C motif chemokine ligand family to be reported (1), and as such, its role during respiratory inflammation or infections is not well understood. We should note that bioinformatics analyses of public gene expression databases indicate that CXCL17 is the highest expressed chemokine in the normal trachea and bronchus and among the highest expressed chemokines in the normal human lung (2). These observations strongly suggest important homeostatic functions for CXCL17 in the respiratory tract. Previous studies have shown that CXCL17 is strongly upregulated in idiopathic pulmonary fibrosis (2). This, along with data from our current study indicates that CXCL17 is very likely to have important functions in the pathogenesis of inflammatory/infectious diseases of the lung as well. Past studies have provided indirect evidence about a possible role of CXCL17 in immunity against respiratory infections. For instance, it has been shown that CXCL17 has a potent bactericidal activity over bacteria causative of respiratory infections (2). The expression of the *CXCL17* gene was also found upregulated in group 3 innate lymphoid cells isolated from lung tissues of patients with tuberculosis (TB) (15), suggesting a role for CXCL17 against *Mycobacterium tuberculosis* (Mtb) infection. However, CXCL17 plays redundant activities during anti-Mtb immunity in murine models (16). In this context, our study represents the first direct evaluation of the expression of CXCL17 during respiratory infections in humans.

Our analyses of lung autopsy specimens suggest that the influenza A(H1N1) pdm09 virus and SARS-CoV-2 stimulate the local production of CXCL17 in the pulmonary tissue. Indeed, although these findings were made only in two patients who died of influenza and two with COVID-19, the pattern of expression observed during both infections differ from what it is observed at steady state. In this regard, using non-infected human lung specimens, we previously demonstrated that CXCL17 is detected only at the luminal surface of alveolar epithelial cells (2). Conversely, here we found that lungs infected with influenza and SARS-CoV-2 express CXCL17 in the whole cytoplasm of pneumocytes, as well as in infiltrating macrophages. This is further supported by a recent paper demonstrating a high induction of CXCL17 in the BAL of COVID-19 (17). However, we found that the magnitude of CXCL17 expression in the serum was also robust during influenza but minimal in COVID-19 patients. These are contrasting observations, although we must consider that the lung tissue specimens in our study were obtained post-mortem. Therefore, our immunohistochemical analysis evaluated the expression of CXCL17 only during the latest phases of viral infection, whereas serum chemokine determinations captured early responses against COVID-19 and influenza. Analysis of CXCL17 expression in lung biopsy specimens obtained early during the infection would have clarified this point. Despite this, we speculate that lung and serum CXCL17 levels may increase as the infection with SARS-CoV-2 progresses in patients with severe disease. Whether longitudinal changes in serum CXCL17 levels could have a prognostic value in COVID-19 should be investigated in future studies.

Our findings may be also explained by possible different abilities of influenza and SARS-CoV-2 virus strains to induce local CXCL17 production and translocation to the blood according to their virulence. Indeed, previous research showed that the infection of human bronchial epithelial cells, human tracheobronchial epithelial cells, and human alveolar A549 cells with seasonal influenza A(H3N2) virus, promotes robust upregulation of *CXCL17*. However, the infection with more virulent A(H5N1) and A(H7N9) influenza virus subtypes induces minor changes in the expression of *CXCL17* (18). Similarly, in a yet unpublished experiment available from the Influenza Research Database (IRD), the infection of 2B-4cells/sorted Calu-3 cells with a wild type strain of the 2002-2003 SARS-CoV (icSARS CoV) did not stimulate strong expression of *CXCL17* (19). In contrast, the infection with icSARS ExoNI and icSARS dNSP16, which are attenuated mutant strains of SARS-CoV, upregulated the expression of *CXCL17*.

Notably, we also demonstrated that these differences could be harnessed for clinical applications, as serum CXCL17 levels determined at hospital admission are useful to distinguish between influenza and COVID-19 in patients with ARDS. This is important, especially at settings of high circulation of pandemic influenza A(H1N1) virus strains, as it is highly likely that both diseases will converge during the upcoming flu season. During such a predicted scenario, the differentiation of influenza and COVID-19 by clinical characteristics may be complicated. Indeed, our results and previous studies show that only a few non-specific symptoms and routine laboratory tests are useful for this diagnostic dilemma (20–22). However, the discrimination of the causative pathogen has direct therapeutic implications, including the selection of the adequate anti-viral drug. Thus, novel biomarkers with high diagnostic value to distinguish influenza and COVID-19 are urgently needed.

Another striking finding of our study is that serum levels of CXCL17 impact on pandemic influenza A(H1N1)-associated mortality. In this sense, it is known that several chemokines participate in the immune response against influenza viruses (23, 24). Most of them are produced in high amounts and mediate pathology due to their pro-inflammatory properties (25, 26). Our data indicate that the production of CXCL17 is also highly potentiated during the early stages of pandemic influenza A(H1N1), as the serum levels reported here are as high as those found in other inflammatory and human autoimmune disorders (2, 11). Moreover, we demonstrate that both macrophages and lung epithelial cells can become sources of CXCL17 after infection with the influenza A(H1N1) pdm09 virus.

CXCL17 normally mediates the recruitment of myeloid cells to the lung (4). Cell subtypes responding to this chemokine include DCs, monocytes, and macrophages (1, 4, 27). However, we should note that the nature of the myeloid cells recruited by CXCL17 *in vivo* has not been established in humans, although the fact that CXCL17 is a mucosal chemokine expressed only in the respiratory and digestive tracts suggests that it is recruiting unique population(s) of myeloid cells to mucosal tissues which remain to be functionally characterized. Increased and sustained recruitment of myeloid cells to the lungs is associated with immunopathology and mortality in influenza (23, 24). Accordingly, our data indicate that influenza patients with higher CXCL17 levels have lower survival, suggesting a possible pathogenic role for this chemokine. We should also mention that CXCL17 also mediates anti-inflammatory activities (25, 26). Thus, our findings may reflect a regulatory mechanism for the cytokine storm underlying severe influenza, *via* the production of high levels of anti-inflammatory mediators.

The role of innate immune cells responding to CXCL17 in COVID-19 is unknown, but recent studies have found that the numbers of circulating DCs and monocytes are reduced in patients with severe SARS-CoV-2 infection as compared to patients with milder forms of the disease (28). In contrast, we found a high number of macrophages in the lung autopsy specimens of patients that died of COVID-19. Together, these data point to a possible role for chemokines involved in the recruitment of myeloid cells to the lungs during COVID-19, and CXCL17 is an excellent candidate to mediate this recruitment (4). However, whether CXCL17 plays a pathogenic or protective role during influenza or COVID-19 is not apparent from our data.

A caveat of our study is that CXCL17 levels were assessed only in influenza patients with severe pandemic influenza A(H1N1), but not in individuals with milder forms of the disease nor in persons with seasonal influenza infections. Hence, future studies must validate our results in other cohorts of influenza patients. Also, we measured serum levels of CXCL17 in COVID-19 patients only at the time of hospital admission. Thus, the analysis of additional time points is required to draw conclusions on the prognostic role of this chemokine in COVID-19. Finally, our findings in PTB patients indicate that CXCL17 is not highly released from the lung to the serum during Mtb infection. This, along with previous results from our group (16), may indicate a minimal role for this chemokine during PTB. Nonetheless, more studies are necessary to rule out a contribution of CXCL17 to the immunity against Mtb.

## Conclusions

In conclusion, our study provides preliminary evidence supporting a diagnostic potential of CXCL17 to distinguish severe pandemic influenza A(H1N1) from COVID-19 and other respiratory infections like PTB. In addition, we show that this chemokine may be useful as a prognostic biomarker in influenza patients, as serum levels of CXCL17 are associated with higher risk of renal failure and mortality. Finally, we found some possible cellular sources of CXCL17 during influenza. Future studies are required to better understand the function of this chemokine in lung defenses against the influenza A(H1N1) pdm09 virus and better evaluated a possible participation in immunity against SARS-CoV-2.

## Data Availability Statement

The raw data supporting the conclusions of this article will be made available by the authors, without undue reservation.

## Ethics Statement

The studies involving human participants were reviewed and approved by IRB of the Instituto Nacional de Enfermedades Respiratorias Ismael Cosío Villegas and Instituto Nacional de Ciencias Médicas y Nutrición Salvador Zubirán. The patients/participants provided their written informed consent to participate in this study.

## Author Contributions

Collected clinical data and biological samples for the study: JC-P, MS-V, CH-C, EH-M, MC-C, NR-Z, CM-M, AC-L, AD, LM-H, DD-Z, DR-G, TR-R, DC-R and CM-M. Performed *in vitro* infections: GR-M, LJ-Á, CC-G, EM-G, and AC-L. Performed CXCL17 ELISA assays: JC-P, LJ-Á, GR-M, and LF-L. Obtained and analyzed autopsy lung specimens: CS-L, CS, FB-M, PG-O, and CL-R. Analyzed and discussed data: JC-P, E-CP, MS-V, EG-L, TR-R, SK, and JZ. Drafted the manuscript: JC-P, SK, AZ, and JZ. All authors contributed to the article and approved the submitted version.

## Funding

Institutional research funds of INER supported the current study. This project also received funding from the National Council of Science and Technology of Mexico (CONACyT) under the research contracts: SECTEI/050/2020, Secretaría de Ciencia, Tecnología e Innovación de la Ciudad de México (SECTEI CDMX); FORDECYT/10SE/2020/05/14-06, CONACYT-Support for scientific research, technological development and innovation in health during COVID-19 contingency, with the project number: 313517, and FORDECYT/10SE/2020/05/14-07 from the Fondo Institucional de Fomento Regional para el Desarrollo Científico y Tecnológico y de Innovación (FORDECYT). JC-P was supported by a scholarship (CVU 737347) from CONACyT to his Ph.D. degree. Funders did not play any role in the study design and conduction.

## Conflict of Interest

The authors declare that the research was conducted in the absence of any commercial or financial relationships that could be construed as a potential conflict of interest.
