# Corrigendum: CXCL17 Is a Specific Diagnostic Biomarker for Severe Pandemic Influenza A(H1N1) That Predicts Poor Clinical Outcome

**DOI:** 10.3389/fimmu.2021.700716

**Published:** 2021-05-13

**Authors:** Jose Alberto Choreño-Parra, Luis Armando Jiménez-Álvarez, Gustavo Ramírez-Martínez, Montserrat Sandoval-Vega, Citlaltepetl Salinas-Lara, Carlos Sánchez-Garibay, Cesar Luna-Rivero, Erika Mariana Hernández-Montiel, Luis Alejandro Fernández-López, María Fernanda Cabrera-Cornejo, Eduardo Misael Choreño-Parra, Alfredo Cruz-Lagunas, Andrea Domínguez, Eduardo Márquez-García, Carlos Cabello-Gutiérrez, Francina Valezka Bolaños-Morales, Lourdes Mena-Hernández, Diego Delgado-Zaldivar, Daniel Rebolledo-García, Parménides Guadarrama-Ortiz, Nora E. Regino-Zamarripa, Criselda Mendoza-Milla, Ethel A. García-Latorre, Tatiana Sofía Rodríguez-Reyna, Diana Cervántes-Rosete, Carmen M. Hernández-Cárdenas, Shabaana A. Khader, Albert Zlotnik, Joaquín Zúñiga

**Affiliations:** ^1^ Escuela Nacional de Ciencias Biológicas, Instituto Politécnico Nacional, Mexico City, Mexico; ^2^ Laboratorio de Inmunobiología y Genética, Instituto Nacional de Enfermedades Respiratorias “Ismael Cosío Villegas”, Mexico City, Mexico; ^3^ Facultad de Estudios Superiores Iztacala, Universidad Nacional Autónoma de México, Mexico City, Mexico; ^4^ Departamento de Neuropatología, Instituto Nacional de Neurología y Neurocirugía “Manuel Velasco Suarez”, Mexico City, Mexico; ^5^ Department of Pathology, Instituto Nacional de Enfermedades Respiratorias “Ismael Cosío Villegas”, Mexico City, Mexico; ^6^ Tecnologico de Monterrey, Escuela de Medicina y Ciencias de la Salud, Mexico City, Mexico; ^7^ Posgrado en Ciencias Biológicas, Universidad Nacional Autónoma de México, Mexico City, Mexico; ^8^ Department of Virology, Instituto Nacional de Enfermedades Respiratorias Ismael Cosío Villegas, Mexico City, Mexico; ^9^ Subdirection of Surgery, Instituto Nacional deEnfermedades Respiratorias Ismael Cosío Villegas, Mexico City, Mexico; ^10^ Departments of Dermatology and Education, Instituto Nacional de Ciencias Médicas y Nutrición Salvador Zubirán, Mexico City, Mexico; ^11^ Centro Especializado en Neurocirugía y Neurociencias México (CENNM), Mexico City, Mexico; ^12^ Departamento de Fibrosis Pulmonar, Instituto Nacional de Enfermedades Respiratorias “Ismael Cosío Villegas”, Mexico City, Mexico; ^13^ Department of Immunology and Rheumatology, Instituto Nacional de Ciencias Médicas y Nutrición Salvador Zubirán, Mexico City, Mexico; ^14^ Respiratory Critical Care Unit, Instituto Nacional de Enfermedades Respiratorias Ismael Cosío Villegas, Mexico City, Mexico; ^15^ Department of Molecular Microbiology, Washington University School of Medicine in St Louis, St. Louis, MO, United States; ^16^ Department of Physiology & Biophysics School of Medicine, Institute for Immunology, University of California, Irvine, CA, United States

**Keywords:** influenza A(H1N1), SARS-CoV-2, COVID-19, tuberculosis, chemokines, CXCL17

An author name was incorrectly spelled as Tatiana Sofia Rodiguez-Reyna. The correct spelling is Tatiana Sofía Rodríguez-Reyna.

In the published article, there was also an error in the affiliation of Criselda Mendoza-Milla. Instead of “Laboratorio de Inmunobiología y Genética, Instituto Nacional de Enfermedades Respiratorias “Ismael Cosío Villegas”, Mexico City, Mexico”, it should be “Departamento de Fibrosis Pulmonar, Instituto Nacional de Enfermedades Respiratorias “Ismael Cosío Villegas”, Mexico City, Mexico”.

The authors apologize for these errors and state that they do not change the scientific conclusions of the article in any way. The original article has been updated.

