# Supplementary material for: CXCL17 Is a Specific Diagnostic Biomarker for Severe Pandemic Influenza A(H1N1) That Predicts Poor Clinical Outcome
Source: Front Immunol. 2021 Feb 26;12:633297. doi: 10.3389/fimmu.2021.633297 (PMC7953906; doi:10.3389/fimmu.2021.633297)
Supplement: Supplementary file 1 [file DataSheet_1.docx]

Supplementary Material

# Supplementary Tables

| Supplementary Table 1. Primer and probe sequences for the detection of SARS-CoV-2 by RT-PCR. | | |
| --- | --- | --- |
| Assay/use | **Oligonucleotide** | **Sequence^a^** |
| RdRP gene | RdRp_SARSr-F | GTGARATGGTCATGTGTGGCGG |
|  | RdRp_SARSr-P2 | FAM-CAGGTGGAACCTCATCAGGAGATGC-BBQ |
|  | RdRP_SARSr-P1 | FAM-CCAGGTGGWACRTCATCMGGTGATGC-BBQ |
|  | RdRp_SARSr-R | CARATGTTAAASACACTATTAGCATA |
| E gene | E_Sarbeco_F | ACAGGTACGTTAATAGTTAATAGCGT |
|  | E_Sarbeco_P1 | FAM-ACACTAGCCATCCTTACTGCGCTTCG-BBQ |
|  | E_Sarbeco_R | ATATTGCAGCAGTACGCACACA |
| N gene | N_Sarbeco_F | CACATTGGCACCCGCAATC |
|  | N_Sarbeco_P | FAM-ACTTCCTCAAGGAACAACATTGCCA-BBQ |
|  | N_Sarbeco_R | GAGGAACGAGAAGAGGCTTG |
| ^a^W is A/T; R is G/A; M is A/C; S is G/C. FAM: 6-carboxyfluorescein; BBQ: blackberry quencher. | | |

| Supplementary Table 2. Clinical characteristics and laboratory parameters of COVID-19 and influenza patients according to their disease outcome. | | | | | | |
| --- | --- | --- | --- | --- | --- | --- |
| Characteristic | **Influenza** | | | **COVID-19** | | |
|  | **Survivors**  **N = 52** | **Deceased**  **N = 16** | ***p*-value** | **Survivors**  **N = 14** | **Deceased**  **N = 10** | ***p*-value** |
| Age (years), median (range) | 48 (20-57) | 49 (37-75) | 0.5397 | 54 (28-71) | 48 (36-73) | 0.9889 |
| Males | 37 (71.1) | 11 (68.7) | >0.9999 | 10 (71.4) | 8 (80) | >0.9999 |
| BMI | 33.4 (30-38.1) | 33.3 (29.7-44.4) | 0.7603 | 28.5 (25.3-30.1) | 29.6 (24.8-31.4) | 0.7961 |
| Comorbidities  Smoking  Diabetes  SAH  OSA  COPD | 19 (36.5)  10 (19.2)  13 (25)  2 (3.8)  1 (1.9) | 8 (50)  4 (25)  5 (31.2)  2 (12.5)  2 (12.5) | 0.3889  0.7255  0.7472  0.2334  0.1357 | 2 (14.2)  5 (35.7)  5 (35.7)  0 (0)  1 (7.1) | 3 (30)  2 (20)  0 (0)  0 (0)  0 (0) | 0.6146  0.6529  0.0530  >0.9999  >0.9999 |
| Symptoms at onset  Fever  Myalgia  Arthralgia  Headache  Dyspnea  Nasal congestion  Rhinorrhea  Sore throat  Thoracic pain  Cough  Sputum  Dry cough  Fatigue  Diarrhea  Nausea  Vomit | 47 (90.3)  44 (84.6)  41 (80.3)  26 (50)  49 (94.2)  7 (13.4)  18 (34.6)  22 (43.1)  6 (11.5)  50 (96.1)  29 (55.7)  20 (39.2)  37 (71.1)  4 (7.6)  2 (3.8)  2 (3.8) | 15 (93.7)  12 (75)  12 (75)  7 (43.7)  16 (100)  6 (37.5)  8 (50)  2 (12.5)  3 (18.7)  16 (100)  9 (56.2)  7 (43.7)  12 (75)  1 (6.2)  2 (12.5)  0 (0) | >0.9999  0.4564  0.7395  0.7776  0.3362  0.0630  0.3783  0.0372  0.4299  >0.9999  >0.9999  0.7742  >0.9999  >0.9999  0.2334  >0.9999 | 11 (78.5)  10 (71.4)  8 (57.1)  6 (42.8)  6 (42.8)  0 (0)  2 (14.2)  2 (14.2)  0 (0)  12 (85.7)  2 (14.2)  10 (71.4)  10 (71.4)  3 (21.4)  3 (21.4)  3 (21.4) | 7 (70)  8 (80)  8 (80)  5 (50)  10 (100)  1 (10)  1 (10)  2 (20)  0 (0)  9 (90)  0 (0)  9 (90)  8 (80)  2 (20)  0 (0)  1 (10) | 0.6653  >0.9999  0.3875  >0.9999  0.0064  0.4167  >0.9999  >0.9999  >0.9999  >0.9999  0.4928  0.3577  >0.9999  >0.9999  0.2391  0.6146 |
| Illness onset - hospital admission  (days) | 7.5 (5-12) | 7.5 (6-11) | 0.9513 | 5.5 (3-11) | 6 (5-11) | 0.4776 |
| Vital signs at admission  Body temperature (^o^C)  Respiratory rate (bpm)  Hearth rate (bpm)  MAP (mmHg) | 38 (37-38)  25 (20-30)  96 (85-109)  86.1 (75-93.5) | 38 (37-38)  24 (20-27)  98 (88-104)  82.8 (74.1-96.5) | 0.9196  0.4427  0.7069  0.9344 | 37 (36.6-37.6)  24 (20-26)  88 (81-99)  81 (75-87.2) | 37 (37-37)  24 (22-26)  78 (69-88)  71.5 (69.5-88.5) | 0.9883  0.9435  0.1185  0.1331 |
| Glucose (mg/dL) | 140 (111.9-207.6) | 179.7 (135.5-277) | 0.0534 | 202 (150.8-281.8) | 124.3 (98.4-179.8) | 0.7521 |
| Blood count  White blood cells (10^9^/L)  Neutrophils (10^9^/L)  Lymphocytes (10^9^/L)  NLR  Hgb (g/dL)  Platelets (10^9^/L) | 7.2 (5.9-22.5)  5.7 (4.6-7.7)  0.8 (0.5-1.1)  8.3 (4.9-12.6)  14.7 (13.2-17.1)  186 (154.3-220.3) | 7.1 (5.1-13.4)  5.8 (4.2-11.4)  0.8 (0.5-0.9)  10.2 (6.9-12.7)  16.4 (14-18.7)  139 (112.3-211.5) | 0.9629  0.7883  0.6951  0.2201  0.1034  0.0342 | 7.7 (4.1-10)  4.7 (2.7-8.9)  0.8 (0.6-1.0)  6.8 (3.2 – 12.5)  14.6 (13.4-16.2)  202 (150.8-281.8) | 9.8 (6.9-14.5)  9.0 (5.9-12.7)  0.8 (0.5-1.2)  12.6 (4.8-17.5)  13.2 (12.2-15.4)  200 (137.8-246.3) | 0.0926  0.0643  0.7409  0.1375  0.0576  0.7961 |
| Renal function  Cr (mg/dL)  BUN (mg/dL)  Na (mmol/L)  K (mmol/L) | 0.9 (0.7-1.3)  20.3 (13.7-33.1)  137.4(133.3-140.5)  4 (3.8-4.3) | 1.1 (0.9-2.3)  29.9 (26.5-54.6)  137.4(134.9-142.2)  4.5 (4-4.7) | 0.1034  0.0014  0.6441  0.0242 | 1.0 (0.8-1.3)  17.5 (13.3-25.9)  137 (135-139.2)  4.1 (3.9-4.3) | 0.9 (0.6-1.5)  20.4 (14.3-32.8)  141.1(138.8-142.4)  4.1 (3.9-4.4) | 0.3948  0.4622  0.0111  0.8744 |
| Liver function  Total bilirubin (mg/dL)  AST (U/L)  ALT (U/L) | 0.5 (0.4-0.8)  60.9 (43-81.8)  37.7 (25.1-51.4) | 0.6 (0.5-0.8)  71.8 (53.4-113.3)  44 (29.9-63.4) | 0.5283  0.0756  0.2042 | 0.4 (0.3-0.7)  32.4 (21.5-46)  29.6 (19.9-41.4) | 0.5 (0.4-0.8)  58.5 (25.5-106.8)  41 (27.3-63) | 0.6559  0.2408  0.1674 |
| Other biomarkers  LDH (U/L)  ALP (U/L)  CPK (U/L)  Procalcitonin (ng/mL) | 616 (461.8-811.1)  118 (98.2-161.1)  274.4 (108-738.6)  0.3 (0.1-1.4) | 800.8 (555.9-1151)  125.1 (97.4-171.1)  248.1 (109.1-506.6)  1.3 (0.4-4.7) | 0.0733  0.5626  0.6444  0.0442 | 289.5(189.5-439.2)  77.9 (69.2-86.8)  83.5 (47.4-429.6)  0.1 (0.05-0.1) | 373 (314.6-504.5)  80.1 (61.3-92.9)  601.3 (69.5-2136)  0.1 (0.08-0.1) | 0.1083  0.8408  0.1721  0.8727 |
| PaO_2_/FiO_2_ | 92.5 (59.9-149) | 76.2 (57.9-129) | 0.4665 | 129.2 (73.9-314) | 113.2 (97.3-172.1) | 0.5233 |
| Severity of illness scores  SOFA  APACHE II | 7 (5-8)  9 (7-15) | 8 (7-12)  14 (9-20) | 0.0369  0.1008 | 3 (2-6)  6 (4-8) | 6 (3-8)  7 (5-12) | 0.0323  0.5932 |
| Respiratory support  High flow nasal cannula  MV  Prone position  ECMO | 0 (0)  52 (100)  30 (57.6)  4 (7.6) | 0 (0)  16 (100)  10 (62.5)  3 (18.7) | >0.9999  >0.9999  0.7797  0.3423 | 7 (50)  7 (50)  3 (21.4)  0 (0) | 0 (0)  10 (100)  5 (50)  0 (0) | 0.0188  0.0188  0.2038  >0.9999 |
| Renal replacement therapy | 5 (9.6) | 11 (68.7) | <0.0001 | 2 (14.2) | 0 (0) | 0.4928 |
| Data are displayed as n (%) or median (IQR). N is the total number of patients with available data. ALP, alkaline phosphatase; APACHE-II, Acute Physiology And Chronic Health Evaluation II; AST, aspartate aminotransferase; ALT, alanine aminotransferase; BMI, body mass index; bpm, breaths/beats per minute; BUN, blood ureic nitrogen; COPD, chronic obstructive pulmonary disease; CPK, creatine phosphokinase; Cr, creatinine; ECMO, extra-corporeal membrane oxygenation; FiO2, fraction of inspired oxygen; HCO3, bicarbonate; Hgb, hemoglobin; IQR, interquartile range; ICU, intensive care unit; LDH, lactate dehydrogenase; MAP, mean arterial pressure; MV, mechanical ventilation; ND, not determined; NLR, neutrophil/lymphocyte ration; OSA, obstructive sleep apnea syndrome; PaO2, partial pressure of oxygen in arterial blood; PCO2, partial pressure of carbon dioxide in blood; SAH, systemic arterial hypertension; SD, standard deviation; SOFA, Sequential Organ Failure Assessment. Differences in continuous variables were estimated using the Mann Whitney U test. Differences in categorical variables were calculated using the Fisher’s exact or the Chi square test as appropriate. | | | | | | |

## Supplementary Figures

**
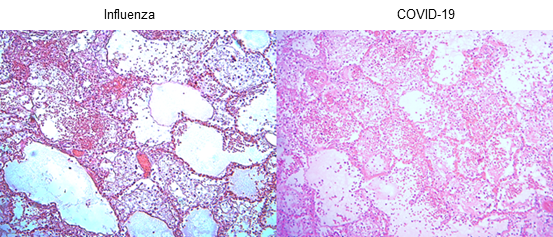
**

**Supplemental Figure 1. Histological characteristics of the lungs of patients with pandemic influenza A(H1N1) and COVID-19.** Lung tissue autopsy specimens were obtained from patients that succumbed to influenza and COVID-19. **Left panel:** Inflammatory infiltrates occupying the alveolar space and composed of macrophages, polymorphonuclear cells, fibrin deposits, as well as hemorrhages were observed in influenza patients. **Right panel:** Morphological changes of COVID-19 consisted of extensive inflammation, thickening of the alveolar walls, and partial loss of the histological architecture. H&E staining, X100.

**Supplemental Figure 2. Diagnostic value of clinical factors and serum CXCL17 levels to distinguish between influenza and COVID-19.** Bivariate logistic regression analysis of the factors predicting the causative pathogen in patients with severe acute respiratory illness. The forest plots show the odds ratio (OR) and 95% CI interval values that were significant for influenza (green squares) and COVID-19 (blue triangles).
